# Patterns of stress and strain in complete-arch prostheses supported by four or six implants: A literature review of finite element analyses

**DOI:** 10.15171/japid.2018.012

**Published:** 2018-12-25

**Authors:** Nasrin Keshavarz Valian, Mohammad Reza Talebi Ardakani, Alireza Aziz Ahari, Mohammad Taghi Baghani, Shireen Shidfar

**Affiliations:** ^1^Department of Periodontics, Dental School, Shahid Beheshti University of Medical Sciences, Tehran, Iran; ^2^Department of Radiology, Rasool-e-Akram Hospital, Iran University of Medical Sciences, Tehran, Iran; ^3^Department of Prosthodontics, Dental School, Shahid Beheshti University of Medical Sciences, Tehran, Iran; ^4^Department of Periodontics, School of Dentistry, Qom University of Medical Sciences, Qom

**Keywords:** All-on-four implant treatment design, all-on-six implant treatment design, finite element analysis, stress

## Abstract

**Background:**

Tilted implants have been recommended as an alternative to the bone graft procedures in implant sites although with possibly higher stress concentrations. This study reviews finite element studies to evaluate patterns of stress and strain in complete-arch prostheses supported by 4‒6 implants.

**Methods:**

A literature search was performed using the online databases. Articles published in English from 2003 to 2015 were reviewed. A total of 100 articles were found related to the subject and after evaluating the titles and abstracts, 18 studies were selected.

**Results:**

By increasing the number of implants, a reduction was detected in the amount of stress in the bone and implants, while in others, the stress level did not change with the increase in the number of implants.

**Conclusion:**

According to finite element analyses, placing a distal implant in an angular position results in better distribution of forces and stresses. Using less cantilever lengths would reduce the stress.

## Introduction

Edentulism is a common condition in elderly patients due to bad oral hygiene, caries and periodontal diseases.^1^ Clinicians are faced with increasing need for a solution for these patients, which is due to increased expectations and lifestyle changes.^2^ These individuals usually have significant bone loss in the alveolar ridge, which might be caused by physiologic or pathologic reasons.^3,4^ A routine treatment for edentulism is the use of conventional dentures while clinical studies comparing patients using dentures with those using implants showed just a slight improvement in quality of life.^5^ These problems and factors such as the predictable results of implant-based prosthetic treatments, awareness of the society and many other factors lead to the increased demands for implants.^6^ Although designing and presenting these treatments require careful attention, cases such as choosing the right area for implant placement, an exact design of the prosthesis, implants’ attachment to each other, their proper diameter and length, the material quality and the prosthesis type in these treatments should be attended carefully to achieve the desired results. In edentulous patients, limitations in the anatomy of the residual bone (such as mandibular canal and maxillary sinus) cause problems in dental implant placement.^7,8^ There are various materials and techniques to overcome this problem.^9,10^ Among these methods, advanced augmentation procedures to achieve adequate bone support for placing standard implants (length: 10‒12 mm and diameter: 3.5 mm) in an extremely atrophic posterior jaw should be mentioned.^11^ However, all of these methods result in the risk of morbidity and complications in patients (such as infection and loss of the graft materials), costs and increased treatment time.^12^

To avoid extensive bone graft procedures and to optimize utilization of the patient's own bone, use of tilted or short implants are alternative methods.^13-19^

Maló et al^23^ introduced (All-on-Four) method. According to this method, four implants will be sufficient for complete restoration of the jaw. Two implants are placed in the anterior areas of jaws and the two others are placed anterior to the mental foramen. Anterior implants are inserted parallel and posterior implants with an approximately 30º distally so that the length of the cantilever is shortened for further prosthetic rehabilitation. By tilting the posterior implant distally, the most posterior placement of the implant is obtained. This permits engagement of implants in the sinus wall and nasal fossa in the maxilla.^20-23^ Nonetheless, according to biomechanical and in vitro studies, tilted implants lead to an increase in the peri-implant bone stress due to bending.^24,25^ These studies were conducted on single implants placed linearly. In contrast, in multiple implants, the rigidity of the prostheses can reduce implant bending. Earlier evaluations have shown that when the posterior implants are placed in a tilted manner, no further loss of bone is detected compared to the parallel ones.^26^

Using four implants for full reconstruction of the maxilla is supported by clinical studies,^27,28^ but it is suggested that use of more implants (about 6) can be safer.^29-31^

Among important biomechanical factors of the implant success, there is stress or the way of force distribution per unit area and strain or amount of change in length per original length when subjected to the applied force. These additional stresses cause extra load and possible failure of implant in future. Stress-related factors can cause complications in short or long term, including implant fracture, crestal bone loss, porcelain fracture, loss of retention, implant component fracture and screw loosening. Therefore, stress distribution is the most important factor, which should be evaluated before the treatment.^32^

In 1976, Weinstein used FEA for the first time in dental implants.^33^ Finite element analysis (FEA) has been extensively utilized in various studies and has been successfully utilized in engineering and biomaterial fields since 1990. FEA includes numerous processes for evaluating structures. Typically, a given topic is much more complicated than can be solved by the usual analysis method. This problem can be stress analysis, heat transfer and large deformations. FEA is based on dividing the problem into smaller and simpler units to find a solution for it.^34^

In the field of implant dentistry, FEA permits calculation of stress distribution in the contact area of the implants with the adjacent bone. However, some assumptions have a major impact on the predictive accuracy of the FEA model. These include model geometry, material properties, applied boundary conditions, and the bone–implant interface. To eliminate these, advanced digital imaging techniques can be utilized.^35^

This study aims at reviewing finite element analysis to assess patterns of stress and strain in complete-arch prostheses supported by 4‒6 implants.

## Methods

An electronic literature search was run using the online databases EMBASE, PubMed and Google Scholar. The following keywords were used to search (in the title and/or in the abstract):

1) "Finite Element" AND "All-on-Four"

2) "Finite Element" AND "All is Four"

3) "Finite Element" AND "All-on-4"

4) "Finite Element" AND "All is 4"

5) "Finite Element" AND "Tilted AND Implant"

6) "Finite Element" AND (Tilted AND Implant)

7) "Finite Element" AND (Tilting AND Implant)

8) "Finite Element" AND (Inclined AND Implant)

9) "Finite Element" AND (Angulated AND Implant)

10) "All is 6" OR "All is Six

Articles in the English language were included. The search included studies involving human subjects and in vitro investigations. No restrictions were employed regarding the design of studies. Additional search was performed through the references of all relevant articles.

The following inclusion criteria were applied: (1) root-form implants were used; (2) some implants were placed in tilted fashion; (3) 4‒6 implants were placed in each jaw; and (4) stress and strain were calculated in peri-implant bone.

When it was necessary, the full texts of the articles were reviewed.

## Results

Articles published in English from 2003 to 2015 were reviewed. A total of 100 articles were found on the subject and after evaluating the titles and abstracts, 18 studies, which were conducted using "Finite Element Analysis" were finally selected. These 18 studies are summarized in Table 1-5 and categorized according to the type of study, date of publication, type of jaw, objectives and the study results.

The articles were examined according to the effects of the number of implants, different angles of implants, cantilever length and stress levels on cortical and trabecular bone.

**Table 1:** Studies included in the review

**Author**	**Study type**	**Date of publication**	**Studied type of jaw**	**Objectives**	**Results**
Watanabe^25^	Finite element analysis (FEA)	2003	Mandible	Evaluation of the stress distribution produced by different degrees of implant body inclination and various positions and loading directions.	Compressive stress levels are higher in the inclined implants. The compressive stress was higher on the cortical bone adjacent to direction of inclination, and tensile stress was pronounced in the opposite direction.
Lan^39^	FEA	2008	Mandible	Evaluation of produced bony stress by different implant tilting during normal masticatory load	Not all types of implant body tilting cause stress concentration.
Bevilacqua^40^	FEA	2008	Mandible	Evaluation of Stress rate around tilted implants against vertical implants	Distal tilted implants in fixed prostheses without cantilever reduce the stress of bone around the implants.
Sasaki^41^	FEA	2008	Mandible	Assessing mechanical risk factors of All-on-Four by evaluating stress distribution on implants and their surrounding bone.	Stress concentration was detected around distal of the tilted posterior implants in the left of jaw. In cases of more spongy bone with less elastic properties, the stress concentration was higher.

**Table 2:** Studies included in the review

**Author**	**Study Type**	**Date of publication**	**Studied type of jaw**	**Objectives**	**Results**
Bellini^42^	FEA	2009	Mandible	Evaluation of stress-induced patterns in cortical bone of mandible with three implant-supported prosthetic designs	There were no significant differences in patterns of stress between tilted implant designs with 5- and 15-mm cantilever. The tilted design of the 15 mm cantilever produced more stress than the 5 mm cantilever.
Bellini^43^	FEA	2009	Maxilla	Evaluation of stress patterns in the implant-bone interface in tilted implant designs and not tilted ones in maxilla	Tilted designs showed a lower amount of compressive stress compared to not tilted ones.
Cruz^44^	FEA	2009	Mandible	Evaluation and comparison of stress distribution around two prosthetic implant systems	Angular systems did not concentrate the stress at any point of the implant. The stress distribution in both systems was very similar to each other.
Silva^36^	FEA	2010	Maxilla	Comparison of the biomechanical behaviour of All-on-Four system with prosthesis supported by implants in Maxilla with tilted dental implants.	Situation patterns and stress distribution were comparable between the two different models. Increasing number of implants reduces the highest stress levels of Von Mises and cantilever increases the stress significantly.

**Table 3:** Studies included in the review

**Author**	**Study type**	**Date of publication**	**Studied type of jaw**	**Objectives**	**Results**
**Bevilacqua** ^45^	FEA	2010	Premaxilla	Comparison and evaluation of the stress transferred to tilted implants against vertical implants and adjacent bone in maxilla	Distal tilted implants, that are hardly splinted by dentures, reduce the stress in the peri-implant bone.
**Takahashi** ^38^	FEA	2010	Mandible	Evaluation of the differences in the stress in the peri-implant cortical bone in models with 6 and 8 implants.	Using 4 implants or inclined implants increases the stress in the peri-implant cortical bone. However, in simultaneous use of short cantilevers, tilting the implants reduce stress in the peri-implant cortical bone.
**Naini** ^46^	FEA	2011	Mandible	Evaluation and comparison of concentration of stress in the peri-implant bone in two designs. 1) design with 4 implants with distal implants of 45 °, 2) 4 parallel implants and vertical to the occlusal plane) and two loading conditions.	None of the designs showed better performance than any of the loading states. Posterior tilted implants were under stress in all situations.

**Table 4:** Studies included in the review

**Author**	**Study type**	**Date of publication**	**Studied type of jaw**	**Objectives**	**Results**
Fazi^37^	FEA	2011	Mandible	Analysis of Stress Distribution in Bone, Implant and Prosthesis in Different Implant Designs in Mandible Implantation	In the designs with parallel implants, 4 and 5 implants cause same stress distribution. Using 4 implants with distal tilted implants of 34-degree caused stress reduction in the bone and implants.
Malhotra^47^	FEA	2012	Mandible	The evaluation of the effect of tilting distal implants at different angles (30 ° and 40°) with different lengths of cantilever (4mm and 12 mm) on the distribution of stress and strain in All-on-Four.	Increasing the angle of the tilted distal implants does not significantly increase the stress level. In addition, the mandible’s structure plays an important role during the treatment planning for complete edentulous patients.
Ozdemir^48^	FEA	2012	Mandible	Evaluation of the force effect on implant and bridges in All-on-Four and alternate designs	In the presence of vertically atrophied mandible although (All-on-Four) method is a clinically possible method, short implants reduce the amount of transferred stress to the supportive bone. The concentration of stress in the cortical bone was greater than the spongy bone. The highest amount of stress in the peri-implant cortical bone was located at distal. By reducing the number of implants, no reduction the plan success rate was observed.

**Table 5:** Studies included in the review

**Author**	**Study type**	**Date of publication**	**Studied type of jaw**	**Objectives**	**Results**
Baggi^49^	FEA	2013	Maxilla-Mandible	Comparing two different repair methods for complete jaw reconstruction by placement of 4 implants	The distal tilted implants presented a better load transfer compared to vertical implants, although the calculated stress in both conditions was physiologically acceptable.
Hussein^50^	FEA	2013	Mandible	Analysis of the Effects of Different Positions of Anterior Implants in Design (All-on-Four).	More concentration of stress was detected on the loading side nearby the posterior implants. The change in the position of anterior implants in the design (All-on-Four) effects the distribution of strain and stress in all of the designs.
Sannino^51^	FEA	2013	Maxilla	Studying the Biomechanical Behaviour of Supported Prostheses by All-On-Four Method by Comparing Three Degree Tilts in Distal Implants.	Maximum level of stress was permanently detected in the distal implant necks. Amount of stress in distal implants was enlarged in apical direction with tilting.
Seker^52^	FEA	2014	Maxilla	Functional stress analysis around the implants and surrounding tissues in posterior maxillary with grafted and non-grafted sites considering the acceptability of various therapeutic options.	The ability to absorb stress is not adequate and is much less than other supporting tissues. Fixed partial prosthesis using short and wide implants with bicortical stability is the most reasonable method for posterior maxillary area.

### A. Number of implants

Silva et al^36^ showed that stress location and distribution in both models with 4 and 6 implants are similar and increasing the number of implants decreases the highest Von Mises stress. Also in a study by Fazi et al^37^ FEA was used to evaluate various implant designs of mandibular fixed prostheses. In this study in which the number of implants varied from 3 to 5, it was shown that implants with parallel position in all the designs exhibited similar stress distribution.

Takahashi et al^38^ examined the effect of implant number on distribution of stress in cortical bone of the mandible in the All-on-Four method. The results showed that by placing 4 implants, stress in the cortical bone around the implants increases compared to 6 implants.

### B.Implant angles

In a study by Lan et al,^39^ the provided stress due to different angles of implant placement was studied. Implants were placed with angles of zero or 15 degrees facing the mesial and distal aspects. This study showed that under horizontal and vertical forces, the highest compressive stresses were located in the cortical bone around the ​implant neck and the results suggested that not all of the tilted implants restored with splinted crowns displayed concentration of stress.

## Discussion

All-on-Four method was first introduced by Malo et al.^20^ As mentioned, based on this concept, 4 implants will be sufficient for complete jaw reconstruction. From these 4 implants, 2 will be placed in the anterior of the alveolus and 2 will be inserted in the anterior area of the mental foramen with a 30° angle toward the distal aspect.

In a clinical study by Malo et al,^23^ complete reconstruction of a jaws supported by 4 implants was evaluated. This retrospective study included 165 complete reconstructions that were immediately loaded and were assessed for 5 years. Fifty-five patients were in the double jaw reconstruction group and 55 patients were in the single jaw reconstruction group. The primary results included cumulative survival of prosthesis and implant and the secondary findings included marginal bone level after 5 years and biological and mechanical complications.

According to the results, one-arch or double-arch restoration in edentulous patients did not display a significant difference in terms of the survival rate.

Malo et al^20^ evaluated the method of All-on-Four for complete reconstruction of edentulous mandible in another clinical study. The results were evaluated clinically for 7 years and radiographically for 5 years. Prosthetic survival rate was 99.7% and the implant’s cumulative survival rate was 95.4%. Therefore, the high survival rate of the prosthesis and implant and the high marginal bone level confirmed the safety of the treatment plan (All-on-Four).

According to the article series presented in Table 1 and the above-mentioned clinical studies, it seems that All-on-Four method is a safe modality of complete reconstruction of the jaw.

Takahashi et al^38^ declared that using angular implants increased stress in the peri-implant cortical bone. However, using these implants in association with a short cantilever reduced stress in the peri-implant cortical bone. Baggi et al^49^ examined two models of full-jaw restoration using 4 implants. In the first technique, two implants were placed vertically at mesial aspect and two posterior implants were inserted with a tilt of 30°, and in the second model every 4 implants were placed with no tilt and in platform-switch fashion. The results of this study confirmed that prostheses which were supported by tilted distal implants showed a more efficient and monotonous load distribution than the vertical position of all the implants. Moreover, distal tilted implants reduced the stress between the bone and the implant in the distal region, but they could cause high tensile stress in the distal crest depending on the bone shape and the force type.

In another study, Bevilacqua et al^40^ compared the stress transmitted to the bone around the implant in the maxilla in tilted and vertical implants. In the developed models, the distal implants were located at angles of 0, 15, 30 and 45 degrees. According to the results, the highest level of stress in the peri-implant bone was recorded for vertical implants (75 MPa) (for implants at the distal aspect) and the lowest for implants at the mesial aspect (35 MPa). Distal tilted implants that had been hardly splint together by fixed denture reduced the stress in the peri-implant bone.

Sannino et al^51^ used FEA to evaluate the All-on-Four Model, using three different degrees (15°, 30° and 45°) in the distal implants. According to the results, there were no significant differences between the 15° and 30° models in terms of Von Mises stress values and the 45° model was the riskiest model in terms of stress in the bone surrounding the implant. The maximum stress was mostly focused in the neck area of the distal implants and the stress in these implants increased in the apical direction with an increase in angle. Bellini et al^43^ compared the produced stress of tilted implant designs in tilted and non-tilted ones in the maxillary bone. According to the results of this study, the tilted designs had less compressive stress compared to non-tilted models.

### C. The length of the cantilever

In a study by Silva et al,^36^ the produced stress pattern in implants in prostheses supported by 4 and 6 implants were compared. In this evaluation, the models were subjected to four loading conditions. The results indicated that in the presence of cantilever, Von Mises stress levels increased 100% in both models. In Bellini's study,^43^ implant-supported prosthetic designs for mandible reconstruction using tilted and non-tilted implants were compared in terms of the stress levels. The first model included 4 implants, among which the distal implants were tilted and two different lengths of cantilever (5 mm and 15 mm) were used for each of the models. The third model consisted of 5 implants which were normally placed and the cantilever length in this case was 15 mm. The results showed that in the tilted model with the 5-mm cantilever and in non-tilted model, the maximum compressive stress level was observed adjacent to the neck of the distal implant. A greater level of compressive stress was inspected near the neck area of the distal implant in the tilted design model with a 15-mm cantilever.

Bevilacqua et al^40^ evaluated the force transfer, using different angles of the implant and different cantilever lengths and the results showed that tilted implants that were splinted in complete fixed prostheses without cantilever caused a reduction in the stress levels in the bone around the implant compared to vertical implants and the cantilever segments.

In a study by Malhotra et al,^47^ the force transfer was evaluated in tilted implants with different lengths of cantilever in All-On-Four position. In these models, the distal implants were tilted in different angles (30° and 40°) and different lengths of the cantilever (4 mm and 12 mm) were used. The results of this evaluation indicated that by increasing the angle in tilted distal implants, the stress levels do not increase significantly. In addition, there was no significant difference between the stress levels and the strain in 4- and 12-mm cantilevers in both positions of the distal implants (30° and 40°).

### D. Stress level in the cortical and trabecular bone

Ozderuir et al^48^ evaluated All-on-Four and alternative designs using FEA. Four models were assessed in this study:

First model: Implants were inserted according to All-on-Four design.

Second model: Two long implants (13 mm in length and 4 mm in diameter) and two short implants (7 mm in length and 4 mm in diameter).

Third model: 4 long implants and two short implants.

Fourth model: Two long implants and four short implants that were inserted vertically.

According to the results of this study, the stress concentration in the neck of the implant and in the cortical bone was significantly higher than the trabecular bone.

## Conclusion

In terms of the implant number in some studies, it has been observed that by increasing the number of implants, a reduction will occur in the amount of stress in the bone and implant, while in others, the stress level did not change by increasing number of implants. In addition, regarding the different angles of implant placement, it seems that placing distal implants in an angular position results in better distribution of the force and stress. However, in some studies it has been mentioned that with increasing the angle, the stress on the peri-implant cortical bone would increase.

In terms of the cantilever length, it seems that using less cantilever lengths would reduce the stress.

## Authors’ contributions

NKV designed the study and proposed preparation and acquisition of data. MRTA and AAA revised the work. MTB acquired data and revised the work. SS interpreted data and drafted the work and finally approved it

## Competing interests

The authors declare no competing interest.

## Ethics approval

There is no need for ethical approval for review articles.
